# Distributed Humidity Sensing in Concrete Based on Polymer Optical Fiber

**DOI:** 10.3390/polym13213755

**Published:** 2021-10-29

**Authors:** Xin Lu, Konstantin Hicke, Mathias Breithaupt, Christoph Strangfeld

**Affiliations:** Bundesanstalt für Materialforschung und-Prüfung, Unter den Eichen 87, 12205 Berlin, Germany; konstantin.hicke@bam.de (K.H.); mathias.breithaupt@bam.de (M.B.); christoph.strangfeld@bam.de (C.S.)

**Keywords:** distributed fiber sensor, fiber optics, material moisture, optical time domain reflectometer, embedded humidity sensors

## Abstract

We present a preliminary investigation on distributed humidity monitoring during the drying process of concrete based on an embedded polymer optical fiber (POF). The water dissipated into the POF changes several properties of the fiber such as refractive index, scattering coefficient and attenuation factor, which eventually alters the Rayleigh backscattered light. The optical time domain reflectometer (OTDR) technique is performed to acquire the backscattered signal at the wavelengths 650 nm and 500 nm, respectively. Experimental results show that the received signal increases at 650 nm while the fiber attenuation factor clearly increases at 500 nm, as the concrete dries out. In the hygroscopic range, the information retrieved from the signal change at 650 nm agrees well with the measurement result of the electrical humidity sensors also embedded in the concrete sample.

## 1. Introduction

Humidity is an important parameter that affects many aspects of our daily life, so the humidity information needs to be monitored and various humidity sensors have been proposed [1]. Humidity measurements based on optical fibers have attracted much interest in recent years thanks to some intrinsic properties of optical fibers, such as small size, chemical inertness, immunity to electromagnetic interference, etc. Particularly, an optical fiber itself can act as a sensing and transmission medium. Optical fiber sensors thus can operate remotely. Moreover, thousands of fiber sensors can be multiplexed due to the large bandwidth of the optical fiber. Optical fiber-based humidity sensors have progressed very fast during the past years and currently, the humidity information can be obtained from the fiber bending loss, absorption loss, or humidity induced strain [2,3,4,5,6]. Special fiber structures, such as fiber tapers, waist-enlarged bitapers and long-period gratings, have been used to improve the response [7,8,9].

In some applications such as structural health monitoring, humidity needs to be monitored over the hygroscopic range. Furthermore, based on these measurements, important material parameters can be derived, such as the diffusion coefficient and the hydraulic conductivity [10]. Distributed fiber sensing is a technique that fulfills this requirement. Several fiber-based distributed humidity sensors have so far been proposed, adapting discrete sensors to a spatially resolved manner. For example, an optical time domain reflectometer (OTDR) can be used to measure the humidity-induced bending loss over a certain distance [3]. The distributed strain sensing technique can also be used to acquire the humidity information by measuring humidity-induced strain in the fiber [5,6]. The reported response of such a sensor can reach up to 38.5 με for 1% relative humidity change, though at the expense of the response time [6]. This type of sensor has also been applied to warn of corrosion under insulation by measuring the water ingress [11]. Note that references [5,6] reveal that fibers with a standard acrylate coating suffer from humidity-induced strain. This can cause errors for other fiber measurements, particularly for distributed acoustic sensing [12] which demonstrates very high sensitivity. Both methods, however, have some drawbacks that prevent them from wide applications. The former requires some water-swellable polymer along the sensing fiber, which is difficult to implement in practice, whereas the latter is essentially based on a distributed strain sensor, whose interrogator is very complex. As a result, the community requires a simple and economic sensing system. Furthermore, the fiber and its coating must withstand the high pH values in concrete of up to 13.5 [13,14].

Most optical fibers are made of silica in order to minimize transmission loss for long-distance communication. However, silica is a chemically inert material, so silica fibers are actually not a good candidate for chemical sensing and various transducers are needed [2]. Like silica fibers, polymer optical fibers (POFs) have been widely used in telecommunication [15,16,17], but they can also be a good solution to chemical sensing because most polymers can absorb chemicals easily. Various chemical sensors based on POF have been proposed and demonstrated [18]. Recently, a POF has been employed to realize distributed humidity sensing based on the OTDR technique [19]. Most POFs can absorb/desorb water and the existence of water in the fiber core can change several fiber properties, for example, the backscattering coefficient, the refractive index and the attenuation coefficient. Some of the property changes can manifest themselves in changes in Rayleigh backscattered light. Liehr et al. [19] report a ~0.5 dB power increase of the backscattered light while the relative humidity (RH) decreases from 90% RH to 30% RH. The Brillouin frequency of perfluorinated POF has been shown to be dependent on the relative humidity, unveiling another way for distributed humidity sensing [20]. However, Brillouin scattering is very weak and difficult to measure in POF. In comparison, OTDR is probably the simplest spatially resolved method to collect Rayleigh backscattered light. As a result, the application of OTDR using POF delivers a simple and economical solution to the challenge of distributed humidity sensing.

Thanks to the fast development in the past few years, the suitability of POF-based point sensors has been demonstrated for a broad range of applications. For example, POFs have been applied to monitor sleep performance, structural health and environmental conditions, respectively [21,22,23]. However, the application of POF-based distributed sensors is rare.

In this paper, relative humidity levels during the drying process of concrete are monitored by a POF sensor using the OTDR technique. Backscattered light at 500 nm and at 650 nm wavelengths, respectively, are obtained and analyzed. Based on power changes of the detected light, the internal RH information is determined, with the obtained values being very close to the results measured by an embedded array of capacitive humidity sensors. This is the first practical application of POF-based distributed humidity sensing to the best of our knowledge, and the obtained results reveal a promising potential for this method to be widely implemented in practice.

## 2. Materials and Methods

### 2.1. Materials

**Figure 1:**
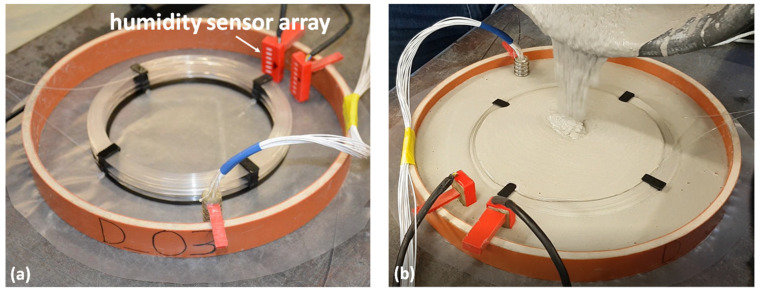
Humidity sensor arrays and the sensing fiber (**a**) before and (**b**) during concrete filling.

The sensing fiber is a ~85 m long POF (Mitsubishi’s Eska GK-40), consisting of a 980 μm poly(methyl methacrylate) (PMMA) core and a fluorinated polymer cladding of 10 μm thickness. The transmission loss of the fiber is ≤150 dB/km at 650 nm and the minimum bending radius is 20 mm. The fiber is divided into five sections as listed in Table 1. Two sections at the near- and far-end of the fiber, respectively, are placed in water to work as a reference. The middle part of the POF is used for material humidity sensing and is coiled in three layers at different heights so that the humidity conditions at various depths of the concrete can be monitored simultaneously. The diameter of each fiber layer ranges from 16 cm to 20 cm. Note that the distance listed in Table 1 is a very conservative estimation, just to make sure the signal from each section range is at the same layer. The casing of the concrete sample is a polyvinyl chloride sewage pipe with an inner diameter of 300 mm as shown in Figure 1a.

The used building material is Knauf FE 50 Largo, representing a common commercial building product. The material was purchased as bagged cargo from a local construction market. The binder and the aggregates were already premixed as dry material. It is a calcium-sulfate-based concrete with a designed compressive strength of 25 N/mm^2^ and an optimal water demand of 0.163 L/kg. Using this water to dry material ratio should result in the building material class A1 [24]. The maximum diameter of the aggregates is below 4 mm, thus, in Germany, it might be classified as screed as well.

To monitor the internal RH of the concrete, the construction material is poured into the casing as shown in Figure 1b and submerges the fiber loops. After the concrete filling, the height of the three layers from the bottom of the concrete is 11 mm, 23 mm and 29 mm, respectively. The total sample height is 35 mm. Two arrays of humidity sensors have also been used to measure the humidity at different depths in order to compare the results with the fiber optic sensor [25]. They are placed at the border of the concrete and a few cm away from the POF, as shown in Figure 1.

In the workshop, the dry material was mixed with tap water by means of an electric hand mixer for three minutes. Then, the entire sample was cast with this mixture. After concreting, the sample was stored in the workshop overnight at around 21 °C. Thereby, the sample was covered by a polyethylene foil with approximately a 2 cm distance to the surface. This after-treatment reduces the evaporation rate and ensures a high humidity above the sample surface. On the next morning, the hardened sample was placed in a climate chamber where the temperature and relative humidity were maintained as 23 °C and 50% RH, respectively. Thus, the monitoring of the hydration and moisture evaporation was started around 16 h after concreting the sample.

### 2.2. Impact of Water on Fiber Property

Water is usually believed to have an obvious influence on the performance of optical fibers made of both glass and polymer, and the influence on POFs is more obvious because the water intake by a polymer can be much larger than by glass. It has been experimentally demonstrated that poly(methyl methacrylate) (PMMA) can absorb ~2% of water by weight [26]. The absorbed water can change the polymer property in two ways. One part of the moisture enters the polymer network and contributes to a volumetric change, and the other part accommodates in microvoids [27].

Several fiber properties can be changed because of water adsorption. According to the Lorentz-Lorenz equation, the refractive index *n* of a polymer with absorbed water can be expressed as

$$\frac{n^{2}-1}{n^{2}+2}=k_{p}\rho_{p}\left(1-f\frac{C_{m}}{\rho_{m}}\right)+k_{m}C_{m}$$

where *ρ* represents the density, *k* is the molar refraction over the molecular weight, *C* is the water concentration, *f* is a parameter about the fraction of absorbed water that contributes to volumetric change and the subscripts *p* and *m* denote for polymer and water, respectively [28]. A reported experiment has shown that the refractive index of PMMA increases for high RH values at room temperature [28].

The light scattering coefficient of the polymer is changed as well due to the water absorption. There are normally many microvoids in the polymer which can deflect the incident light. However, 40% to 60% of the absorbed water inside PMMA accommodates in these microvoids [27], which is supposed to alleviate the light scattering. Therefore, the backscattering might be weakened due to water adsorption in the POF.

The influence of water on fiber attenuation is evident and well-known. The molecular vibration of aliphatic hydrocarbons is normally the main light loss source for PMMA polymer, and the harmonics of the carbon–hydrogen (CH) absorption defines several transmission channels in PMMA POF [29]. However, absorbed water inside the POF can cause extra optical attenuation at certain wavelengths due to the oxygen-hydrogen (OH) vibrational absorption. In the visible spectral range, the OH absorption peak is supposed to appear at 520 nm, 562 nm, 614 nm and 674 nm, corresponding to v_6_OH, v_5_OH + δOH, v_5_OH and v_4_OH + δOH, respectively, where v*_n_*OH represents *n*-th harmonic of OH stretching vibration and δOH is the OH bending vibration [30].

The analysis above reveals that many properties of the polymer can be changed by the water absorption and a combination of the property variations can modify the light propagating inside the POF. For example, the light at 650 nm experiences more loss in more humid environments while the fiber attenuation becomes lower at 500 nm under the same condition [30]. However, current theoretical analysis usually focuses on one aspect, making it difficult to describe the scattering process inside the fiber core. Consequently, there lacks a theoretical model to predict the relationship between the backscattered light level and the relative humidity change to the best of our knowledge.

The influence of water absorption on the fiber property can thus be exploited for humidity sensing. The power level of the backscattered light is observed to shift under different humidity conditions, and distributed humidity sensing has been realized by collecting the light backscattered from a POF using the OTDR technique [19]. The same method is used here to monitor the RH in concrete during its drying process by embedding the fiber into the material. Note that some elements may diffuse from the surrounding concrete into the POF, and thus may change the fiber properties and the Rayleigh backscattered light.

### 2.3. Methods

To realize distributed humidity sensing, the backscattered light from the fiber was acquired in a spatially resolved manner by the OTDR technique. Such an OTDR system sends short optical pulses into the fiber and collects the backscattered light continuously along the fiber. The spatial resolution was determined by the pulse length and the distance can be calculated by the time-of-flight of the optical pulse. A commercial photon-counting OTDR system (Luciol L220 POF) was used here, which exhibits good performance detecting weak signals. To further improve the quality of the obtained OTDR trace, the signal was averaged for 1 h for each measurement. The sampling interval of the trace was 2.5 cm. During the measurement, the OTDR traces at 500 nm and 650 nm were acquired alternately.

## 3. Results and Discussion

The measurements at the two wavelengths exhibit distinct behaviors during the concrete drying process, the level of the backscattered light at 650 nm increases during the measurement, whereas the slope of the OTDR trace, that is, the fiber attenuation coefficient, obtained at the other wavelength changes obviously as the concrete dries. Hence, the measurements at the two wavelengths are analyzed separately in this section.

### 3.1. Results at 650 nm

**Figure 2:**
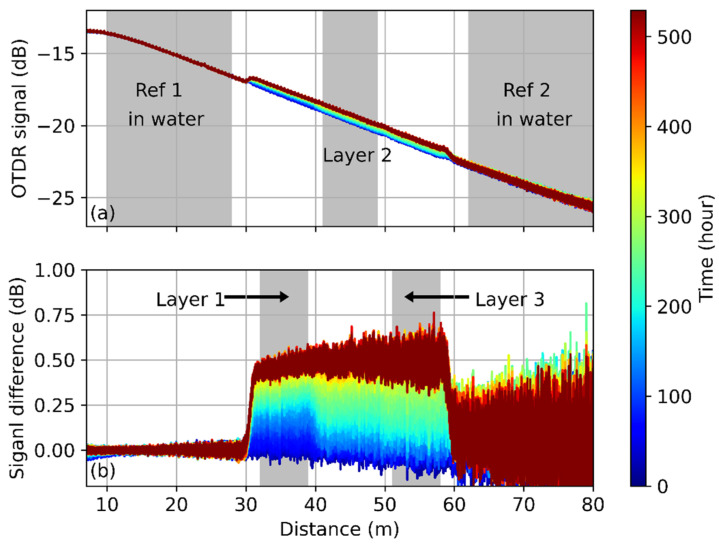
OTDR measurement at 650 nm during the concrete drying process. (**a**) Obtained OTDR traces and (**b**) OTDR signal changes compared with the first trace. Trace color denotes the measured time.

The OTDR traces obtained at 650 nm are plotted in Figure 2a, and the trace color represents the measurement time. The reference sections at the near- and far-end, respectively, are immersed in water, so the signal from these positions remains very stable during the whole sample drying. At the beginning of the measurement, the concrete is very moist, and water penetrates into the POF. According to Figure 2a, the signal obtained at the sensing part increases with time because water is released from the sample. In order to focus on the signal change, Figure 2b plots the OTDR trace difference from the first measurement result. It shows more clearly that the signal in Ref. [1] changes little during the measurement, but the signal varies significantly in Ref. [2] at the far end of the fiber, which can be explained by the low SNR. At the sensing section, the signal difference becomes larger as the concrete dries out as expected. It is interesting to note that the difference at Layer 1 grows faster than other layers. This layer is at a higher position, closer to the interface of concrete and air, so the water evaporates faster than at the deeper part.

**Figure 3:**
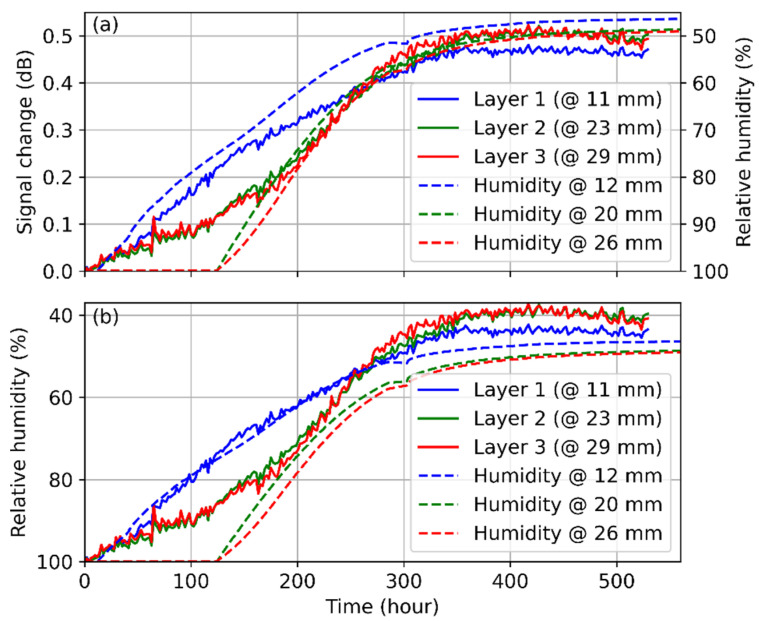
Comparison of the results obtained by fiber optic and electrical humidity sensors at different levels. (**a**) Temporal evolution of the OTDR signal change (*y*-axis on the left-hand side) vs. the relative humidity measured by the sensor array (*y*-axis on the right-hand side). (**b**) Comparison of the relative humidity measured by the fiber optic and humidity sensors. Solid and dashed lines denote the results of fiber optic and electrical sensors, respectively.

According to the analysis in Section 2.2, the absorbed water changes the POF properties, which may result in a lowered scattering coefficient. As a result, the power level of the backscattered light is low at the beginning of the measurement when the concrete is moist but increases as the water evaporates from concrete to air. The OTDR traces shown in Figure 2 agree with this analysis, implying that the humidity can be calibrated from the power level of the detected light. To calibrate relative humidity, the averaged signal difference at each layer is plotted in Figure 3 and compared with the result obtained by the humidity sensors at a similar depth. The blue line in the figure represents the result at the top of the concrete, it is higher than the other lines from 0 h to ~280 h. This behavior agrees with common sense, in that the top surface dries faster than the other parts. Furthermore, all the measured results seem to level out after 300 h, as the internal humidity of the concrete becomes similar to the ambient environment. However, the signal change at the lower level (red and green solid lines) is larger at this stage, which can be explained by the OH absorption at ~650 nm. As the water dissipates into the air, the extra loss induced by OH becomes smaller, so the signal increases during the measurement. Since the fiber attenuation is accumulated along the fiber, the signal change at the far-end (i.e., at the lower level) is more obvious.

Distributed humidity sensing based on POF is at its very preliminary stage, there lacks analysis on the conversion of the signal change into relative humidity values. According to Figure 3a, the backscattered signal increases by ~0.41 dB while the RH drops from 100% to 50% at 11 mm. It is very similar to the result in [19], the backscattered signal changes about 0.5 dB as the RH decreases from 90% to 30%. The change is almost linear for both cases. Thus, a conversion ratio of 0.0083 dB/% RH is obtained between the signal change and the RH variation and is used to retrieve the humidity information from the OTDR signal change shown in Figure 3a. The result is presented in Figure 3b and is compared with the RH measured by the electronic sensor. The solid and dashed blue lines correspond to the obtained humidity information at ~11 mm, and they overlap until 300 h, indicating a good agreement between the two sensor types. This confirms the functionality of the POF as a humidity sensing medium. However, a deviation is observed after 300 h, which can be explained by the fiber attenuation change.

At the two greater depths of 23 mm and 29 mm, the humidity sensors are saturated for the first 120 h. This is expected behavior because after concreting, the pore system is over-saturated as well. Due to hydration and evaporation, pore saturation decreases until the hygroscopic range of <96% RH is reached. At around 80% RH, the fiber and the humidity sensors highly correlate. It is noticeable, that the signal amplitude of the fiber at 23 mm and 29 mm already decreases during the first 120 h. This indicates that the fiber is able to measure the material humidity in the over-hygroscopic range as well. This gives further possibilities of application in the field of water engineering and soil physics. However, the OTDR signal at Layer 2 and 3 shows an RH value lower than the ambient condition (50%). A possible explanation is that the fiber sections absorb some other substance from the concrete during the drying process, so the backscattering is enhanced. It may also be explained by the inaccurate conversion ratio under this condition.

### 3.2. Results at 500 nm

**Figure 4:**
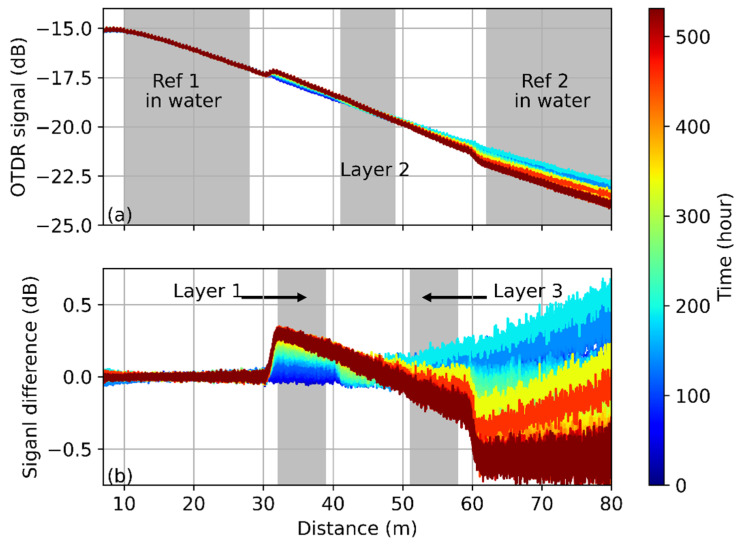
OTDR measurement at 500 nm during the concrete drying. (**a**) Obtained OTDR traces and (**b**) OTDR signal changes compared with the first trace. Trace color denotes the measured time.

The OTDR traces obtained at 500 nm are shown in Figure 4a and they are affected by the water-induced variations of both, the scattering coefficient and fiber attenuation, respectively, as discussed above. While the concrete hydrates and dries out, the slope of the trace becomes steeper. This behavior indicates the fiber attenuation gets larger and it is quite different from the case at 650 nm, in which the change of scattering coefficient dominates the process. This tendency agrees with the results reported in Ref. [30], which experimentally demonstrates that the water absorbed in the POF can reduce the fiber loss at 500 nm. Figure 4b shows the trace change compared with the first obtained OTDR trace. The plotted curves are the result of a joint impact of the scattering coefficient and fiber attenuation change. At the beginning of the sensing section, the detected signal is more influenced by the scattering coefficient change induced by the adsorbed water, it thus increases as the internal RH drops. According to Figure 4, the change of scattering coefficient and fiber attenuation have similar but contrary impacts on the obtained signal at ~ 46 m, and they cancel each other out. Hence the signal change at that position is almost null during the measurement. The fiber attenuation change dominates after this point, so the obtained signal decreases as the RH drops instead of increasing.

The combined impact of the changing scattering coefficient and the fiber attenuation change makes it difficult or even impossible to retrieve the RH information based on a single parameter. For example, the signals received at layer 1 and layer 3 change in opposite directions as the humidity decreases. It is thus necessary to calibrate the RH based on the variations of both parameters in the future.

It has to be noted that the humidity-induced fiber attenuation is more significant in the concrete measurement at hand than in the results reported in Ref. [19]. This may be explained by the ambient conditions. In Ref. [19], the sensing fiber was placed in a climate chamber, so that the fiber is only subject to the humidity change and the impact of other factors is highly suppressed. However, various kinds of elements exist in the concrete, some of them may diffuse into the fiber core during the measurement and change the fiber property. This finding indicates that the sensing fiber needs to be well protected in practice in order to ensure that the fiber is sensitive only to the target chemical.

## 4. Conclusions

This paper presents a preliminary exploration of distributed humidity sensing inside a concrete sample with an embedded PMMA POF being employed as the sensing medium. We believe it is the first application demonstration of POF-based distributed humidity sensing. The absorbed water changes the light guiding property of the fiber so that the humidity information can be acquired in a spatially resolved manner based on the measurement of Rayleigh backscattered light. The RH retrieved from the OTDR signal at 650 nm agrees with the results obtained by the embedded capacitive humidity sensor array, validating the feasibility of the distributed fiber sensor based on POF. The measurement shows that the top and middle of the material dries faster, which agrees with common sense. However, the results obtained here are different from those in Ref. [19], probably due to the sorption of other elements from the construction material.

Although the obtained results validate the functionality of the POF sensor under practical conditions, some issues need to be addressed to facilitate wide application. For example:

1. The impact of the humidity on the fiber property needs to be further investigated to develop a quantifiable relationship between the RH change and changes of the backscattered light.
2. The sensor should be highly selective to a particular analyte, in this case, water. A protective coating or jacket is necessary to allow the target to reach the fiber and block other elements.

## Figures and Tables

**Table 1:** Information about different fiber sections.

	Ref. [1]	Layer 1	Layer 2	Layer 3	Ref. [2]
Distance (m)	10–28	32–39	41–19	51–58	62–85
Surrounding	Water	Concrete	Concrete	Concrete	Water

## Data Availability

The data presented in this study are available on request from the corresponding author.
